# Histoplasma capsulatum Infection With Both Granulomatous Features and Fibrosing Mediastinitis Presenting as Shortness of Breath: A Case Report

**DOI:** 10.7759/cureus.70398

**Published:** 2024-09-28

**Authors:** Ryan M Kozloski, Alec K Donohue, Preston J Sparks, Janelle L Robertson, David C Hostler

**Affiliations:** 1 Internal Medicine, Womack Army Medical Center, Fort Liberty, USA; 2 General Surgery, Womack Army Medical Center, Fort Liberty, USA; 3 Thoracic Surgery, Archbold Medical Center, Thomasville, USA; 4 Infectious Disease, Womack Army Medical Center, Fort Liberty, USA; 5 Pulmonary and Critical Care Medicine, VA Coastal Health Care System, Fayetteville, USA

**Keywords:** asthma, fibrosing mediastinitis, granulomatous disease, hilar fullness, interdisciplinary, lack of response to therapy, video assisted thoracoscopic surgery

## Abstract

It is known that Histoplasma capsulatum can cause chronic granulomatous disease or fibrosing mediastinitis, but both presentations occurring in the same patient is exceedingly rare and difficult to diagnose. The patient is a 24-year-old female with a past medical history of asthma, who presented for worsening shortness of breath. Thoracic imaging revealed a large paratracheal mass with a significant mass effect. Endobronchial ultrasound (EBUS) was attempted unsuccessfully, and the patient underwent video-assisted thoracoscopic surgery, which established the diagnosis of fibrosing mediastinitis. Extensive infectious disease workup confirmed Histoplasma infection. She was treated with a course of Itraconazole, pulmonary artery stenting, and rituximab.

## Introduction

Fibrosing mediastinitis due to histoplasmosis is a well-described, albeit rare, outcome of infection with the fungus Histoplasma (H.) capsulatum and complicates an estimated 3 in 100,000 infections. Mediastinal granulomas are also a known but rare complication of H. capsulatum infection. It is typically thought that these presentations represent different dysregulated host immune responses, but they have not previously been described as occurring in the same patient. The clinical presentation is variable, with symptoms including dyspnea, hemoptysis, or dysphagia [1-3]. Successful diagnosis and treatment require a high index of suspicion and, often, a multispecialty approach.

This article was previously presented as a meeting abstract at the 2022 CHEST Annual Scientific Meeting on October 17, 2022.

## Case presentation

**Figure 1:**
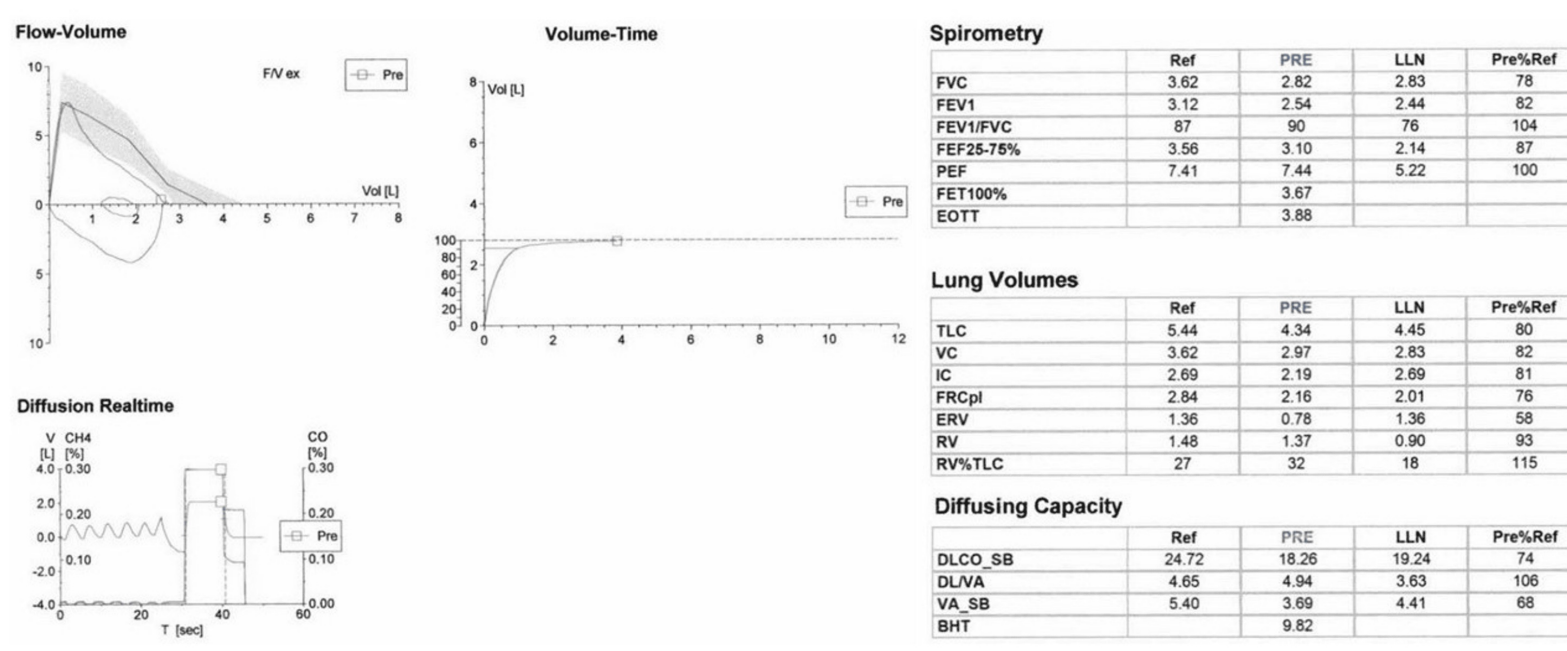
Pulmonary function testing data Baseline spirometry identified an isolated decreased FEV1 with a significant bronchodilator response which normalized airflow. There was a mild restrictive defect by plethysmography and a Mild reduction in DLCO. F/V ex: flow rate of expiration divided by volume of air expired, PRE: prebronchodilator testing, VOL: volume, V: volume, T: time, CH4: methane, CO: carbon monoxide, Ref: age, gender, and size-controlled reference values, LLN: lower limit of normal, Pre%Ref: percentage of reference value from prebronchodilator testing, FVC: forced vital compacity, FEV1: forced expiratory volume exhaled in the first second, FEF25-75%: forced mid-expiratory flow, PEF: peak expiratory flow, FET100%: forced expiratory time, EOTT: end of test time, TLC: total lung compacity, VC: vital compacity, IC: Inspiratory compacity, FRCpl: functional residual compacity, ERV: expiratory reserve volume. RV: residual volume, DLCO_SB: diffusion compacity of the lungs for carbon monoxide, VA_SB: alveolar volume, BHT: breath hold test

A 24-year-old female, with a history of asthma, presented for shortness of breath, orthopnea, and dyspnea on exertion refractory to daily controller medications and albuterol. She grew up in Memphis, Tennessee, where she had been diagnosed with exercise-induced asthma as a teenager and was prescribed an inhaler. However, over the last few months, she developed rapidly worsening symptoms and was unable to walk up a low incline without significant shortness of breath. She was trialed on daily fluticasone propionate/Salmeterol as well as an albuterol rescue inhaler without improvement in her symptoms. Physical exam and initial laboratory workup were unrevealing. Pulmonary function testing (Figure 1) showed a mildly reduced forced expiratory volume in one second (FEV1), which resolved with a bronchodilator, restricted lung volumes, and a small decrease in diffusion compacity of the lungs for carbon monoxide, which did not seem to explain the severity of her symptoms.

**Figure 2:**
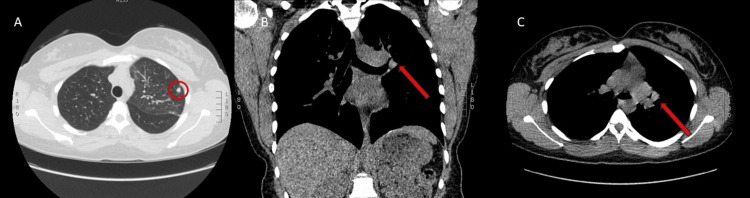
Computed tomography scan A. Axial image demonstrating a 7-millimeter upper lobe nodule. B and C. Coronal and axial images showing perihilar fullness concerning for mediastinal adenopathy and large soft tissue mass in the left paratracheal space with extrinsic compression of the mass on the left main bronchus.

Chest X-ray showed prominence of the left hilum and a follow-up CT scan (Figure 2) revealed both perihilar fullness concerning for mediastinal adenopathy and a large soft tissue mass in the left paratracheal space with extension into the hilum. The mass was exerting mass effect on the left main bronchus, left upper lobe bronchus, and left pulmonary artery. There was also a discrete 7 mm upper lobe nodule.

**Figure 3:**
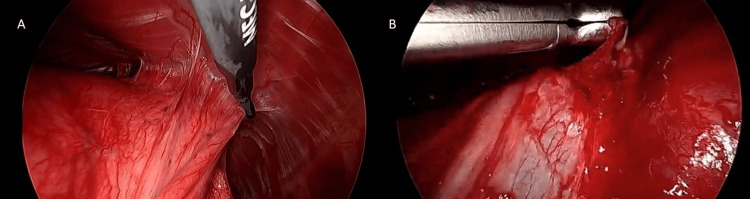
Images from diagnostic VATS A. Extensive adhesions were found and lysed. B. Scanlan forceps were used to biopsy the mass in the hilum. VATS: video-assisted thoracoscopic surgery

This raised concern for malignancy, and the patient proceeded to endobronchial ultrasound (EBUS). On white-light bronchoscopy, the left-sided airways showed hypervascularity and extrinsic compression of the airways beginning just proximal to the left upper lobe takeoff and extending down to the left upper lobe airways. The bronchoscope could not be advanced into the segmental airways of interest, but EBUS was able to identify an ill-defined perihilar fullness. Biopsies were attempted, but it was difficult to pass a needle into the firm lesion and minimal paucicellular aspirate was returned. Due to continued diagnostic uncertainty, the patient was referred for a diagnostic video-assisted thoracoscopic surgery (VATS) (Figure 3).

**Figure 4:**
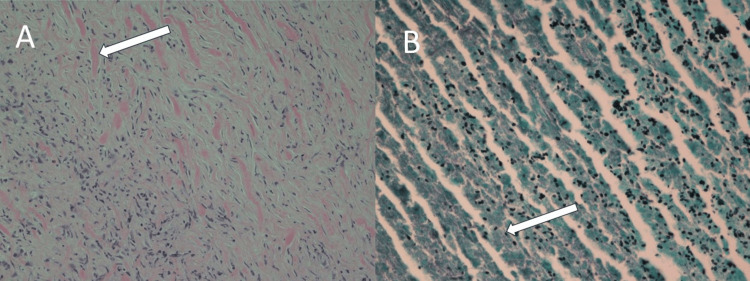
Pathology results A. (Hematoxylin and Eosin 10X): Biopsy of the fibrotic mass in the hilum showed fibrosis infiltrating the mediastinal adipose tissue with thickened collagen and inflammatory infiltrate composed primarily of lymphocytes and plasma cells with rare eosinophils, findings characteristic of fibrosing mediastinitis. B. (Grocott’s Methenamine Silver 40X): Caseating granuloma that highlights abundant yeast-like organisms within the central necroinflammatory debris. These organisms were morphologically consistent with Histoplasma capsulatum.

Thoracoscopic biopsy was performed but was technically difficult due to the fibrotic nature of the tissue. The left upper lobe nodule was removed by wedge resection and appeared grossly to be a caseating granuloma. A biopsy of the fibrotic mass in the hilum (Figure 4) showed fibrosis infiltrating the mediastinal adipose tissue with thickened collagen and inflammatory infiltrate composed primarily of lymphocytes and plasma cells with rare eosinophils, findings characteristic of fibrosing mediastinitis. Pathology of the upper lobe nodule confirmed it was a caseating granuloma with abundant yeast-like organisms within central necroinflammatory debris. These organisms were morphologically consistent with Histoplasma capsulatum.

In the setting of extrinsic compression of the left pulmonary artery to the point of near-obliteration, the patient underwent stent placement in the left pulmonary artery, with complete resolution of her orthopnea and significant improvement of her other symptoms [4,5]. She underwent extensive infectious disease evaluation (Table 1).

**Table 1:** Relevant laboratory data indicating that the patient had either an active Histoplasma infection with a low organism burden or a previous infection causing a delayed immune response AFB: acid-fast Bacilli

Test	Result
AFB culture and smear	Negative X3
Tissue cultures	Negative
Urine Histoplasma antigen	Negative
Histoplasma antibody by immunodiffusion	Negative
Histoplasma M band	Positive at 1:1 dilution
Histoplasma mycelial CF antibody	Positive at 1:2 dilution
Histoplasma yeast CF	Positive at 1:2 dilution

Given the component of granulomatous disease, she was treated with a 12-week course of Itraconazole. Unfortunately, repeat CT imaging showed no improvements. She was then treated with a rituximab infusion followed by a second infusion two weeks later with plans to repeat every six months.

## Discussion

Fibrosing (sclerosing) mediastinitis is an archetypal clinicopathologic diagnosis; to wit, there are no “smoking-gun” histologic findings that enable the diagnosis. Instead, histologic findings compatible with the disease process must be viewed in conjunction with characteristic radiologic and clinical findings to arrive at the correct diagnosis. Most importantly from a pathologic perspective, histologic findings of other disease processes that mimic the radiologic and clinical findings of fibrosing mediastinitis must be excluded to arrive at the correct diagnosis.

In this case, mediastinal biopsies from the radiologically appreciated left hilar mass/mediastinal thickening demonstrated mediastinal lymphoid and fibroadipose connective tissue with infiltrative inflammatory fibrosis. Inflammatory fibrosis consists of a patternless, fibroblastic spindle cell proliferation with abundant intervening collagen deposition and areas of thickened (keloidal-type) collagen. No malignant cytomorphologic features (e.g. necrosis, mitotic figures, marked nuclear atypia) were present within the spindled cells. Interspersed throughout the fibrous proliferation were chronic inflammatory infiltrates composed of abundant lymphocytes and plasma cells with scattered eosinophils. Important pathologic entities to be excluded (as referenced above) based on the radiologic appearance, anatomic location, and histologic appearance include a spindle cell carcinoma arising from the lung, spindled mesothelioma, lymphoma (e.g. nodular sclerosis Hodgkin lymphoma), thymoma, and sarcoma (e.g. inflammatory myofibroblastic tumor). These entities were excluded based on a combination of histologic findings, immunohistochemical stains, and flow cytometric analysis of the sampled tissue.

Given the exclusion of these mimics and histologic findings compatible with fibrosing mediastinitis, the clinical picture becomes essential for the diagnosis of fibrosing mediastinitis. Mediastinal granuloma is the more common condition and occurs due to dysregulated immune response to fungal antigens within the lymph nodes [1]. The nodes enlarge and form a necrotic mass. Fibrosing mediastinitis is a rarer, but well-described disease process, in which the fungal antigens leak into the extracellular space and the immune response induces fibrotic changes within the tissue [2]. This typically arises years after infection in patients who have spent extended periods of time in a Histoplasma endemic region, though many of these patients never have a documented acute Histoplasma infection [1-3]. The fibrosis is progressive and there is no cure. Antifungals are not routinely recommended for fibrosing mediastinitis; however, it is recommended in cases of mediastinal granuloma or in cases that cannot be distinguished between the two [6]. Recent data suggest rituximab limits the progression of fibrosing mediastinitis, though limited data confirm the efficacy of this intervention [7,8]. Additional treatment focuses on addressing the patient’s symptoms caused by local invasion of the fibrosis; in this case, extrinsic compression of the left-sided airways and left pulmonary artery [4,5]. Finally, this patient was from a hyper-endemic area (Memphis, TN) and she was within the typical demographic, given she is a female less than 40. This should have caused increased suspicion of the diagnosis earlier in her course. Given the complexity and rarity of this condition, as well as the uncertainty surrounding the efficacy of treatments, it is important to involve multiple specialties as well as the patient in treatment discussions to ensure that the risks and benefits are fully understood prior to initiating treatment [9].

## Conclusions

This case demonstrates that both fibrosing mediastinitis and granulomatous disease can occur simultaneously in a single patient. Though these are rare and can be difficult to diagnose, they should be considered in patients who do not respond to initial therapy. Further, as these conditions represent two different dysregulated immune responses to the same stimulus it is unclear what the most effective treatment options are. Cases such as this also demonstrate the need for coordination among providers from multiple specialties to provide effective patient-centered care.
